# The impact of diabetes on the relationship of coronary artery disease and outcome: a study using multimodality imaging

**DOI:** 10.1186/s12933-023-01850-3

**Published:** 2023-05-31

**Authors:** Matias Mäenpää, Iida Kujala, Esa Harjulahti, Iida Stenström, Wail Nammas, Juhani Knuuti, Antti Saraste, Teemu Maaniitty

**Affiliations:** 1 Turku PET Centre, University of Turku and Turku University Hospital, P.O. Box 52, 20521 Turku, Finland; 2 Heart Center, Turku University Hospital, Turku, Finland; 3 Department of Clinical Physiology, Nuclear Medicine and PET, Turku University Hospital, Turku, Finland

**Keywords:** Computed tomography angiography, Coronary artery disease, Diabetes, Hybrid imaging, Outcome, Perfusion

## Abstract

**Background:**

Patients with prediabetes or diabetes are at increased risk of developing cardiovascular disease and adverse outcomes. First-line coronary computed tomography angiography (CTA) followed by selective use of positron emission tomography (PET) myocardial perfusion imaging is a feasible strategy to diagnose and risk-stratify patients with suspected coronary artery disease (CAD). The aim of the present study was to study whether diabetes changes the relationship of CAD and long-term outcome.

**Methods:**

We retrospectively identified consecutive symptomatic patients who underwent coronary CTA for suspected CAD. In patients with suspected obstructive CAD on CTA, myocardial ischemia was evaluated by ^15^O-water PET myocardial perfusion imaging. The relationship of the phenotype of CAD and long-term outcome in patients with no diabetes, prediabetes, or type 2 diabetes was investigated. A composite endpoint included all-cause mortality, myocardial infarction (MI), and unstable angina pectoris (UAP).

**Results:**

A total of 1743 patients were included: 1214 (70%) non-diabetic, 259 (15%) prediabetic, and 270 (16%) type 2 diabetic patients. During 6.43 years of median follow-up, 164 adverse events occurred (106 deaths, 41 MIs, 17 UAPs). The prevalence of normal coronary arteries on CTA was highest in the non-diabetic patients (39%). The prevalence of hemodynamically significant CAD (abnormal perfusion) increased from 14% in non-diabetic patients to 20% in prediabetic and 27% in diabetic patients. The event rate was lowest in patients with normal coronary arteries and highest in patients with concomitant type 2 diabetes and hemodynamically significant CAD (annual event rate 0.2% vs. 4.7%). However, neither prediabetes nor diabetes were independent predictors of the composite adverse outcome after adjustment for the clinical risk factors and imaging findings.

**Conclusions:**

Coronary CTA followed by selective downstream use of PET myocardial perfusion imaging predicts long-term outcome similarly in non-diabetic and diabetic patients.

**Supplementary Information:**

The online version contains supplementary material available at 10.1186/s12933-023-01850-3.

## Background

Patients with type 2 diabetes have increased risk of developing coronary artery disease (CAD) [1]. Prediabetes, i.e. a state of impaired glycaemia not fulfilling the criteria for diabetes, is also associated with increased risk of developing cardiovascular disease [2]. Furthermore, type 2 diabetes and prediabetes are associated with impaired survival compared with nondiabetic patients [3, 4].

Coronary computed tomography angiography (CTA) allows non-invasive detection of non-obstructive or obstructive CAD and provides prognostic information. However, coronary CTA is limited in assessing the hemodynamic significance of a coronary stenosis, often requiring further functional evaluation for myocardial ischemia, such as myocardial perfusion imaging (MPI) [5]. Positron emission tomography (PET) enables quantitative MPI to assess the hemodynamic significance of epicardial coronary stenosis as well as coronary microvascular dysfunction, and its use has been rapidly increasing in recent years [6]. Furthermore, combining data from different imaging modalities, such as coronary CTA and PET, may provide incremental diagnostic and prognostic information [7].

Previously, quantitative ^82^Rb PET MPI showed that patients with type 2 diabetes and reduced myocardial flow reserve (MFR) had high annual adverse event rate, whereas diabetic patients with preserved MFR had similar outcome as nondiabetic patients with reduced MFR [8]. Furthermore, Murthy et al. found an extremely low rate of cardiac mortality in diabetic patients with preserved CFR as quantified by ^82^Rb PET [9]. Combination of anatomical and functional information may provide complementary prognostic information [10, 11] but the value of combined coronary CTA and myocardial perfusion imaging in diabetic patients remains uncertain.

In our hospital, selective hybrid CTA/PET imaging is provided as routine clinical service in symptomatic patients with suspected CAD [12]. The hemodynamic significance of suspected obstructive stenosis detected by coronary CTA is routinely evaluated by ^15^O-water PET MPI. We sought to compare imaging phenotypes of CAD and their association with long-term outcomes in patients with no diabetes, prediabetes, or type 2 diabetes.

## Materials and methods

### Patients

From our institutional registry, we identified 2212 consecutive symptomatic patients who underwent clinically indicated coronary CTA due to suspected CAD at the Turku University Hospital during the period from 2008 to 2016. Patients with previously known obstructive CAD (i.e., obstructive stenosis on invasive coronary angiography, prior myocardial infarction, or prior coronary revascularization), or patients undergoing CTA for assessing the etiology of cardiomyopathy or heart failure, were not considered for inclusion. According to the local clinical routine, coronary CTA scan was first performed, and the CTA findings were promptly evaluated by an attending physician [12]. In case of suspected obstructive stenosis on CTA (≥ 50% in diameter), myocardial ischemia was routinely evaluated by ^15^O-water PET MPI during adenosine vasodilation (stress-only protocol) if there were no contraindications.

In the current study, we focused on patients with type 2 diabetes or prediabetes and compared them with non-diabetic patients. Hence, 22 patients with type 1 diabetes, 9 patients with other type of diabetes mellitus (e.g., MODY or LADA), and 245 patients with unknown diabetes status were excluded. Additionally, we excluded 62 patients who did not undergo PET perfusion imaging despite suspected obstructive CAD on coronary CTA, and 128 patients due to non-diagnostic CTA and/or PET imaging results. Lastly, 3 patients were excluded due to unavailable follow-up data. Consequently, the final study cohort consisted of 1743 patients with known diabetes status, fully characterized CAD phenotype by CTA/PET imaging, and complete follow-up data.

### Clinical and follow-up data

Diabetes status, other traditional risk factors for CAD (hypertension, dyslipidemia, smoking, and family history), symptoms, echocardiographic findings (within 6 months prior to imaging), exercise electrocardiography (ECG) findings (within 6 months prior to imaging), and medication use, were retrospectively collected from electronic medical records. Prediabetes was defined as impaired fasting glucose (fasting plasma glucose 6.1–6.9 mmol/l), impaired glucose tolerance (2-h plasma glucose 7.8–11.0 mmol/l in a 75 g oral glucose tolerance test), or hemoglobin A1c 6.0–6.4%/42–47 mmol/mol within 6 months prior to imaging [2]. Type 2 diabetes was defined as prior diagnosis based on medical records, the use of glucose-lowering therapy (excluding off-label use), plasma fasting glucose ≥ 7.0 mmol/l, 2-h plasma glucose ≥ 11.1 mmol/l, or hemoglobin A1c ≥ 6.5%/ 48 mmol/mol [2].

Follow-up data until May 2020 were obtained on all-cause mortality, myocardial infarction (MI), and unstable angina pectoris (UAP) using hospital discharge registry data (Auria Clinical Informatics) and the events were manually confirmed using electronic medical records. In case of multiple events in a single patient, the first one was considered. Information on early (6-month) invasive coronary angiography (ICA) and myocardial revascularization by either percutaneous coronary intervention (PCI) or coronary artery bypass graft surgery (CABG) was also recorded, but these were not considered as adverse events.

### Image acquisition

The coronary CTA and PET imaging procedures have been previously described in detail [12]. Coronary CTA and PET perfusion scans were performed with a 64-row hybrid PET-CT scanner (GE Discovery VCT or GE D690, General Electric Medical Systems, Waukesha, Wisconsin). Prior to coronary CTA, isosorbide dinitrate aerosol or sublingual nitrate was administered. Intravenous metoprolol was administered if needed to achieve target heart rate of < 60 beats/min. Coronary calcium score was measured (in 82.5% of the patients) using the Agatston method. Coronary CTA was performed using intravenously administered low-osmolal iodine contrast agent. Prospectively ECG-triggered acquisition was applied whenever feasible. Dynamic ^15^O-water PET myocardial perfusion scan during adenosine vasodilator stress (140 µg/kg/min) was then selectively performed if obstructive CAD was suspected based on the CTA scan. Coronary CTA and PET perfusion scans were usually performed in the same imaging session, but however, in some patients PET perfusion imaging was performed in the following days or weeks due to logistic reasons or caffeine use.

### Image analysis and interpretation

Coronary CTA scans were analysed using GE ADW Workstation (General Electric Medical Systems, Waukesha, Wisconsin) according to the segmentation system recommended by the SCCT guidelines [13]. A diameter stenosis of ≥ 50% was considered obstructive. Dynamic PET data were analysed using Carimas software (Turku PET Centre, Turku, Finland) in standard 17 myocardial segments model (excluding basal septal segments 2 and 3). Absolute stress segmental (regional) myocardial blood flow < 2.3 ml/g/min was considered abnormal and indicative of myocardial ischemia as previously shown [14]. The analysis and interpretation of imaging data were performed by experienced physicians and recorded in a standardized reporting system.

**Fig. 1:**
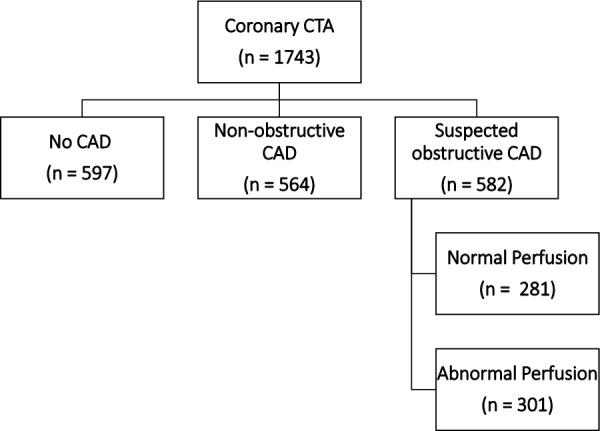
Imaging findings based on hybrid CTA/PET approach

Based on the coronary CTA and stress PET perfusion findings, patients were categorized as having (1) normal coronary arteries, (2) non-obstructive CAD, (3) suspected obstructive CAD but normal myocardial perfusion, or (4) suspected obstructive CAD and abnormal myocardial perfusion (Fig. 1).

### Statistical analysis

Continuous variables are shown as mean ± SD or median (25th and 75th percentile). Categorical variables are shown as counts and percentages. ANOVA (post-hoc Tukey) or Kruskal-Wallis (post-hoc Bonferroni) test was used to compare continuous variables among the groups of non-diabetic, prediabetic, and diabetic patients, whereas categorical variables were compared with chi square test. Survival curves were created based on Kaplan-Meier estimates and were compared with Mantel-Cox pooled log-rank test. Cox proportional hazards model was applied to identify the predictors of composite adverse endpoint of mortality, MI or UAP, and statistical interaction was also tested. Statistically significant univariable predictors (*p* < 0.05) were included in the multivariable analyses. Annual event rates for the composite endpoint were calculated and compared by using Poisson regression. The statistical analyses were conducted with IBM SPSS Statistics version 27.

## Results

Among 1743 patients, 1214 had no diabetes (69.7%), 259 had prediabetes (14.9%), and 270 had type 2 diabetes (15.5%). Patient characteristics are shown in Table 1.

**Table 1:** Clinical characteristics, medication, imaging findings, early invasive procedures, and outcomes during follow-up

	Total cohort	No diabetes (0)	Prediabetes (1)	Type 2 diabetes (2)	Overall p value	p value (0 vs 1)	p value (0 vs 2)	p value (1 vs 2)
N	1743 (100%)	1214 (69.7%)	259 (14.9%)	270 (15.5%)				
Characteristics								
Age (years)	62.0 (± 9.9)	61.5 (± 10.2)	62.6 (± 9.1)	64.0 (± 9.1)	< 0.001	0.202	< 0.001	0.248
BMI	28.0 (± 6.9)	26.7 (± 6.5)	29.6 (± 7.3)	31.5 (± 6.9)	< 0.001	< 0.001	< 0.001	0.023
Male sex	714 (41.0%)	467 (38.5%)	117 (45.2%)	130 (48.1%)	0.005	0.139	0.01	1
Hypertension	996 (57.1%)	594 (48.9%)	180 (69.5%)	222 (82.2%)	< 0.001	< 0.001	< 0.001	0.009
Dyslipidemia	1124 (64.5%)	730 (60.1%)	197 (76.1%)	197 (73.0%)	< 0.001	< 0.001	< 0.001	1
Family history	846 (48.5%)	624 (51.4%)	122 (47.1%)	100 (37.0%)	< 0.001	0.628	< 0.001	0.062
Smoking status					< 0.001	< 0.001	< 0.001	1
Ex-smoker	366 (21.0%)	234 (19.3%)	64 (24.7%)	68 (25.2%)				
Current smoker	213 (12.2%)	123 (10.1%)	44 (17.0%)	46 (17.0%)				
Symptoms					0.326			
Typical angina pectoris	374 (21.5%)	258 (21.3%)	56 (21.6%)	60 (22.2%)				
Atypical chest pain or dyspnea	1152 (66.1%)	792 (65.2%)	173 (66.8%)	187 (69.3%)				
Other	217 (12.4%)	164 (13.5%)	30 (11.6%)	23 (8.5%)				
Exercise ECG finding*, ‡	Available for 1124 (64.5%)	Available for 800 (65.9%)	Available for 172 (66.4%)	Available for 152 (56.3%)	< 0.001	0.069	< 0.001	0.227
Ischemia on exercise ECG	512 (45.6%)	397 (49.6%)	69 (40.1%)	46 (30.3%)				
Left ventricular ejection fraction on echocardiography*, ‡	Available for 914 (52.4%)	Available for 657 (54.1%)	Available for 120 (46.3%)	Available for 137 (50.7%)	0.374			
Reduced ejection fraction (< 50%)	52 (5.7%)	36 (5.5%)	5 (4.2%)	11 (8.0%)				
eGFR (ml/min/1.73m2)	82.4 (± 14.6)	82.5 (± 14.3)	82.1 (± 15.1)	82.7 (± 15.9)	0.680			
Fasting plasma glucose (mmol/L)	6.0 (± 1.1)	5.4 (± 0.5)	6.1 (± 0.4)	7.2 (± 1.6)	< 0.001	< 0.001	< 0.001	0.166
HbA1c (mmol/mol)	39.4 (± 7.6)	36.0 (± 4.2)	40.3 (± 4.2)	49.6 (± 11.6)	< 0.001	< 0.001	< 0.001	< 0.001
LDL cholesterol (mmol/L)	2.9 (± 0.94)	2.9 (± 1.0)	3.0 (± 0.8)	2.4 (± 0.7)	< 0.001	1	0.001	< 0.001
Baseline medications								
Beta-blocker	760 (43.6%)	475 (39.1%)	135 (52.1%)	150 (55.6%)	< 0.001	< 0.001	< 0.001	0.596
Lipid-lowering drug	664 (38.1%)	413 (34.0%)	99 (38.2%)	152 (56.3%)	< 0.001	< 0.001	< 0.001	0.464
Antiplatelet drug	716 (41.1%)	472 (38.9%)	113 (43.6%)	131 (48.5%)	0.001	0.292	0.001	0.329
Anticoagulant	153 (8.8%)	110 (9.1%)	22 (8.5%)	21 (7.8%)	0.911			
Long-acting nitrate	120 (6.9%)	67 (5.5%)	15 (5.8%)	38 (14.1%)	< 0.001	1	< 0.001	< 0.001
ACEi or ARB	656 (37.6%)	372 (30.6%)	121 (46.7%)	163 (60.4%)	< 0.001	< 0.001	< 0.001	0.001
Calcium channel blocker	272 (15.6%)	148 (12.2%)	52 (20.1%)	72 (26.7%)	< 0.001	0.003	< 0.001	0.056
Glucose-lowering agents	222 (12.7%)	3 (0.2%)	1 (0.4%)	218 (80.7%)	< 0.001	1	< 0.001	< 0.001
Insulin	47 (2.7%)	0 (0%)	0 (0%)	47 (17.4%)	< 0.001	1	< 0.001	< 0.001
Imaging findings								
CTA/PET hybrid imaging finding					< 0.001	< 0.001	< 0.001	0.035
Normal coronary CTA	597 (34.3%)	474 (39.0%)	71 (27.4%)	52 (19.3%)				
Non-obstructive CAD	564 (32.4%)	388 (32.0%)	88 (34.0%)	88 (32.6%)				
Suspected obstructive CAD but normal perfusion	281 (16.1%)	176 (14.5%)	47 (18.1%)	58 (21.5%)				
Obstructive CAD and abnormal perfusion	301 (17.3%)	176 (14.5%)	53 (20.5%)	72 (26.7%)				
Global stress myocardial blood flow*	3.10 (± 1.06)	3.04 (± 1.09)	3.33 (± 1.21)	3.15 (± 0.88)	0.515			
Calcium score category*	Available for 1438 (82.5%)	Available for 1016 (83.7%)	Available for 212 (81.9%)	Available for 210 (77.8%)	< 0.001	0.017	< 0.001	< 0.001
0	517 (36.0%)	415 (40.8%)	61 (28.8%)	41 (19.5%)				
1–99	409 (28.4%)	283 (27.9%)	76 (35.8%)	50 (23.8%)				
100–399	282 (19.6%)	194 (19.1%)	38 (17.9%)	50 (23.8%)				
> 400	230 (16.0%)	124 (12.2%)	37 (17.5%)	69 (32.9%)				
Absolute calcium score*	27 (0–215)	11 (0–164)	31.5 (0–167)	197 (16–559)	< .001	0.077	< 0.001	< 0.001
Follow-up								
Early ICA†	205 (11.8%)	115 (9.5%)	33 (12.7%)	57 (21.1%)	< 0.001	0.415	< 0.001	0.008
Early PCI†	101 (5.8%)	57 (4.7%)	18 (6.9%)	26 (9.6%)	0.005	0.476	0.005	0.562
Early CABG†	17 (1.0%)	11 (0.9%)	1 (0.4%)	5 (1.9%)	0.208			
Early PCI OR CABG†	115 (6.6%)	67 (5.5%)	18 (6.9%)	30 (11.1%)	0.004	1	0.002	0.162
Death	106 (6.1%)	64 (5.3%)	12 (4.6%)	30 (11.1%)	0.001	1	0.001	0.006
MI	41 (2.4%)	27 (2.2%)	4 (1.5%)	10 (3.7%)	0.227			
UAP	17 (1.0%)	13 (1.1%)	3 (1.2%)	1 (0.4%)	0.541			
Death/MI	147 (8.5%)	91 (7.5%)	16 (6.1%)	40 (14.8%)	< 0.001	1	0.001	0.004
Death/MI/UAP	164 (9.4%)	104 (8.6%)	19 (7.3%)	41 (15.2%)	0.005	1	0.005	0.02

*BMI* body mass index, *CAD* coronary artery disease, *ECG* electrocardiogram, *eGFR* estimated glomerular filtration rate, *HbA1c* hemoglobin A1c, *LDL* low-density lipoprotein, *ACEi* angiotensin converting enzyme inhibitor, *ARB* angiotensin II receptor blockers, *CTA* computed tomography angiography, *PET* positron emission tomography, *ICA* invasive coronary angiography, *PCI* percutaneous coronary intervention, *CABG* coronary artery bypass graft, *MI* myocardial infarction, *UAP* unstable angina pectoris

^*^Variable not available for all patients. Proportions are calculated among patients with data available

^†^ Early procedures defined as within 6 months after the CTA/PET imaging

^‡^Within 6 months prior the CTA/PET imaging

Patients with type 2 diabetes were older, more frequently male and had less often family history of premature CAD than non-diabetic patients. Non-diabetic patients had lower body mass index and were less often hypertensive, dyslipidemic or smoking as compared with patients having prediabetes or type 2 diabetes. There was no difference in the rate of angina pectoris among the 3 groups, but patients with type 2 diabetes had less frequently ischemia on exercise ECG. Patients with prediabetes or type 2 diabetes were more often using anti-ischemic medication, lipid-lowering medication, antithrombotic medication, and angiotensin converting enzyme inhibitors (ACEi) or angiotensin receptor blockers (ARB), compared with non-diabetic patients.

### Imaging findings

Obstructive CAD was excluded by coronary CTA alone in 1161 (67%) patients (normal coronary CTA in 597 patients and non-obstructive CAD in 564 patients). In turn, 582 (33%) patients underwent PET perfusion imaging for hemodynamic evaluation of suspected obstructive CAD, of whom 281 patients had normal and 301 had abnormal perfusion. The radiation dose was 7.5 ± 3.4 mSv from coronary CTA and 0.96 ± 0.18 mSv from PET perfusion imaging.

**Fig. 2:**
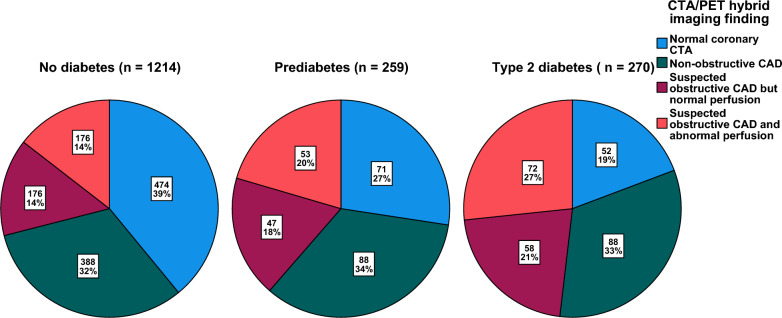
Combined CTA/PET imaging findings according to diabetes status

The prevalence of normal coronary arteries on CTA was highest in the non-diabetic patients (39%). In contrast, the prevalence of hemodynamically significant CAD (abnormal perfusion) increased from 14% in non-diabetic patients to 20% in prediabetic and 27% in diabetic patients (Table 1 and Fig. 2).

Coronary artery calcium score was available for 1438 (83%) patients in the cohort, and the amount of coronary calcification was associated with the diabetes status (Table 1). The prevalence of zero calcium score was 41% in non-diabetic patients and decreased to 29% in prediabetic and 20% in diabetic patients. Conversely, the presence of high (> 400) calcium score was 12% in non-diabetic patients and increased to 17% in prediabetic and 33% in diabetic patients.

### Annual rate of adverse events

During a median follow-up of 6.43 years (25th-75th percentiles 4.63–8.62), 164 adverse events were recorded, including 106 deaths, 41 MIs and 17 UAPs. In 597 patients with normal coronaries on CTA there were 8 adverse events (6 deaths and 2 MIs). In 564 patients with non-obstructive CAD there were 51 adverse events (39 deaths, 8 MIs and 4 UAPs). In 281 patients with suspected obstructive CAD but normal perfusion there were 36 adverse events (19 deaths, 15 MIs and 2 UAPs). In 301 patients with suspected obstructive CAD and abnormal perfusion there were 60 adverse events (33 deaths, 16 MIs and 11 UAPs).

Annual rate of composite endpoint (death/MI/UAP) was 1.33% (95% CI 1.14–1.56%) for the whole study cohort, 1.23% (95% CI 1.01–1.50%) in non-diabetic, 1.02% (95% CI 0.65–1.59%) in prediabetic patients, and 2.16% (95% CI 1.57–2.97%) in patients with type 2 diabetes. Patients with type 2 diabetes had higher event rate than non-diabetic patients (p = 0.003), whereas event rates were similar in prediabetic and non-diabetic patients (p = 0.450).

**Fig. 3:**
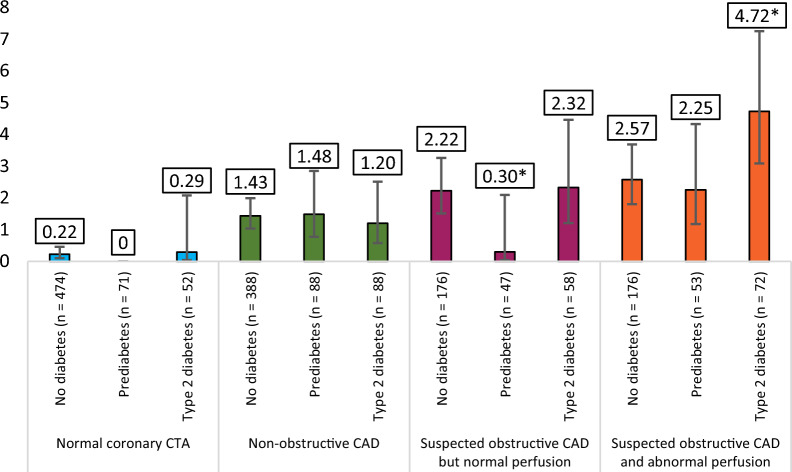
Annual composite adverse event % rates (with 95% confidence intervals) stratified by diabetes status and combined CTA/PET imaging findings. Comprehensive statistics provided in Additional file 1: Table S1. *Indicates statistical significance

**Fig. 4:**
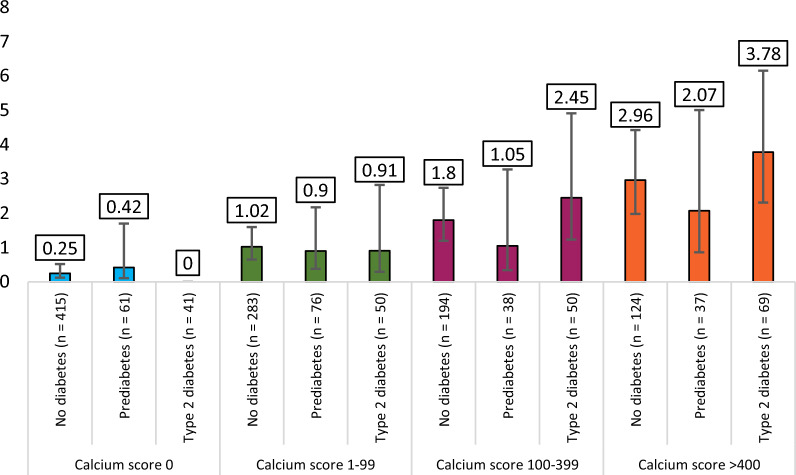
Annual composite adverse event % rates (with 95% confidence intervals) stratified by diabetes status and Agatston calcium score. Comprehensive statistics provided in Additional file 1: Table S2

Figure 3 shows the annual event rate stratified by diabetes status and hybrid CTA/PET imaging findings. Figure 4 shows the annual event rate stratified by diabetes status and Agatston calcium score. The details of the event numbers and rates are shown in Additional file: Tables S1 and S2.

The rate of subsequent early ICA and early revascularization, respectively, after CTA/PET imaging were 0% and 0% in patients with normal coronary CTA, 2.8% and 0.2% with non-obstructive CAD, 6.0% and 1.1% with obstructive CAD but normal PET perfusion, and 57.1% and 37% with abnormal PET perfusion. Patients with type 2 diabetes underwent coronary revascularization more often than non-diabetic patients (11% vs. 6%, p = 0.002).

### Predictors of adverse events

In Cox regression analysis, univariable predictors of adverse events were increasing age, male sex, type 2 diabetes, hypertension, typical angina pectoris, coronary artery calcium score and CTA/PET imaging findings (Table 2). In the multivariable model with hybrid CTA/PET imaging, age, hypertension, and hybrid imaging findings remained independent predictors of events, whereas neither prediabetes nor type 2 diabetes was independent predictor of events (Table 2). Likewise, in the multivariable model with coronary calcium score, age and coronary artery calcium score remained independent predictors of adverse events, whereas neither prediabetes nor type 2 diabetes was independent predictor.

**Table 2:** Cox proportional hazards model predictors for adverse events

Univariable predictors of the composite endpoint (MI/UAP/death)			Multivariable model with CTA/PET imaging		Multivariable model with calcium score	
Model	HR (95% CI)	p-value	HR (95% CI)	p-value	HR (95% CI)	p-value
Age (years)	1.08 (1.06–1.10)	< 0.001	1.07 (1.04–1.09)	< 0.001	1.05 (1.02–1.07)	< 0.001
BMI (kg/m2)	0.98 (0.96–1.00)	0.171				
Male sex	1.40 (1.02–1.92)	0.035	1.27 (0.91–1.79)	0.160	1.20 (0.81–1.77)	0.370
Smoking history	1.36 (0.99–1.87)	0.061				
Diabetes						
No diabetes	Reference		Reference		Reference	
Prediabetes	0.80 (0.49–1.31)	0.377	0.61 (0.37–1.01)	0.053	0.73 (0.42–1.28)	0.270
Type 2 diabetes	1.76 (1.21–2.55)	0.003	1.12 (0.76–1.64)	0.570	1.12 (0.74–1.85)	0.512
Hypertension	2.21 (1.55–3.17)	< 0.001	1.52 (1.04–2.20)	0.029	1.31 (0.85–2.01)	0.222
Dyslipidemia	1.01 (0.73–1.41)	0.94				
Family history of CAD	0.74 (0.54–1.03)	0.07				
Typical angina pectoris	1.49 (1.05–2.14)	0.028	1.23 (0.86–1.77)	0.257	1.28 (0.85–1.93)	0.239
Calcium score						
0	Reference				Reference	
1–99	3.97 (1.87–8.45)	< 0.001			3.01 (1.39–6.50)	0.005
100–399	7.31 (3.50–15.27)	< 0.001			4.59 (2.12–9.96)	< 0.001
> 400	12.38 (6.05–25.32)	< 0.001			6.86 (3.15–14.95)	< 0.001
CTA/PET imaging						
Normal coronary CTA	Reference		Reference			
Non-obstructive CAD	7.17 (3.40–15.11)	< 0.001	4.60 (2.15–9.81)	< .001		
Suspected obstructive CAD but normal perfusion	9.57 (4.45–20.59)	< 0.001	4.89 (2.22–10.75)	< 0.001		
Suspected obstructive CAD and abnormal perfusion	14.92 (7.14–31.21)	< 0.001	8.69 (4.03–18.7)	< 0.001		
Early CABG or PCI	2.14 (1.34–3.43)	0.001				

^*^Abbreviations as in Table 1

Sub-analyses restricted to patients not undergoing early revascularization were carried out for both multivariable models: CTA/PET finding remained an independent predictor of outcome (p < 0.001) while type 2 diabetes did not (p = 0.597). Similarly, calcium score was an independent predictor (p < 0.001) while type 2 diabetes was not (p = 0.833).

There was no significant statistical interaction between diabetes status and CTA/PET imaging findings for predicting composite adverse endpoint (p-value 0.319). Similarly, there was no interaction between calcium score and diabetes status for predicting events (p-value 0.937).

**Fig. 5:**
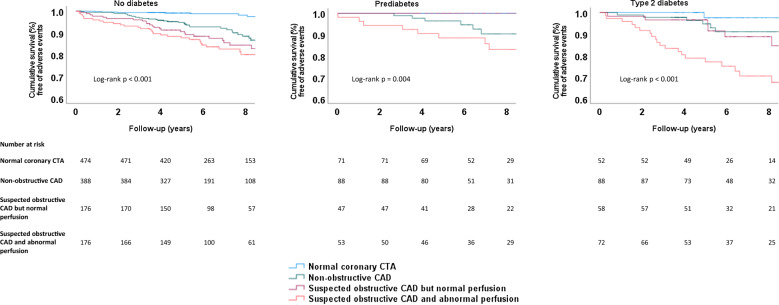
Kaplan-Meier survival curves of CTA/PET findings, stratified by diabetes status

Figure 5 shows Kaplan–Meier survival curves for CTA/PET imaging findings separately for patients with no diabetes, prediabetes, and type 2 diabetes. The most favourable outcome was associated with normal coronary CTA imaging finding, whereas the poorest outcome was associated with obstructive CAD and abnormal perfusion despite diabetes status. Survival between imaging finding groups was statistically different among patients with no diabetes (p-value < 0.001), prediabetes (p-value 0.004), and type 2 diabetes (p-value < 0.001).

## Discussion

Selective combined imaging strategy with initial coronary CTA and downstream PET MPI identifies patients at increased risk for future adverse events among those referred for evaluation of chest pain or equivalent symptoms [12]. The current study demonstrates that advanced anatomical and functional coronary imaging provides comparable prognostic information in symptomatic patients with no diabetes, prediabetes, or type 2 diabetes. The prevalence of obstructive CAD and myocardial ischemia are higher in diabetic patients as expected, and in addition, the risk of death, MI or UAP are higher in these patients. However, there was no significant interaction between the presence of prediabetes/type 2 diabetes and the prognostic value of CTA/PET imaging findings.

The highest annual adverse event rate (up to 4.7%) was found in patients with type 2 diabetes and hemodynamically significant CAD, i.e., obstructive coronary stenosis on CTA and abnormal myocardial stress perfusion on PET. This is in line with previous studies demonstrating high mortality in patients with concomitant diabetes and CAD [15]. Interestingly, previous evidence indicated that the prognosis is comparable for non-diabetic patients with abnormal myocardial flow reserve and diabetic patients with preserved myocardial flow reserve [8, 16]. Moreover, Murthy et al. reported a very low annual rate of cardiac mortality in diabetic patients with preserved CFR whereas diabetics with reduced CFR had annual cardiac mortality rate comparable to patients with prior CAD [9].

In our study, type 2 diabetes was associated with worse outcome (unadjusted HR 1.76, p = 0.003) but was not an independent predictor in multivariable models adjusted for either CTA/PET imaging findings or coronary calcium score, separately. Furthermore, the findings were consistent in sub-analyses restricted to patients not undergoing early revascularization. This may be partly explained by generally low rate of adverse events in the contemporary population such as in our study. However, this is in line with a large cohort study showing that the presence of diabetes was not a predictor of future MI in patients without CAD on coronary angiography, although being associated with all-cause mortality [17]. The absence of significant interaction between diabetes status and the prognostic value of CTA/PET imaging findings suggests that the prognostic performance of CTA/PET imaging was consistent in diabetics, prediabetics, as well as non-diabetic patients.

The current European guidelines recommend the use of coronary CTA especially in symptomatic patients with relatively low pre-test likelihood of CAD and highlight its high negative predictive value both regarding diagnosis and prognosis [5]. In our study cohort the prevalence of hemodynamically significant CAD was almost two-fold (26.7% vs. 14.5%) in type 2 diabetes patients compared with non-diabetic patients, which is in line with previous literature regarding diabetes-related risk of CAD [1]. Importantly, obstructive CAD could be still excluded by coronary CTA alone in about half the diabetic patients in our study cohort, including 19% with normal coronary arteries and 33% with anatomically non-obstructive CAD, and this was associated with low adverse event risk despite the presence of diabetes. The good prognosis of patients without CAD in our study is in line with previous studies evaluating diabetics with the use of coronary CTA [18]. This suggests that coronary CTA can be used as a first-line diagnostic test for suspected CAD in diabetic similarly to non-diabetic patients, and the need for downstream testing for myocardial ischemia remains reasonable.

Both microvascular and macrovascular involvement is well documented in diabetic CAD patients, and worse outcome may in part be attributable to coronary microvascular dysfunction [9, 19, 20]. The treatment options for coronary microvascular dysfunction are limited, further emphasizing the need for strict glycaemic control and other preventive strategies in diabetic patients [21]. Per our institutional protocol, myocardial perfusion imaging is triggered by a suspected obstructive stenosis on coronary CTA, and therefore, “pure” coronary microvascular dysfunction without CAD may be missed. On the other hand, in patients undergone PET perfusion imaging, quantification of stress myocardial blood flow integrates the effects of both epicardial and microvascular coronary circulation.

In our study cohort, the outcome of prediabetic patients was quite similar to nondiabetic patients. However, prediabetic patients with obstructive CAD on CTA but normal myocardial perfusion had unexpectedly low rate of adverse events. We acknowledge that this may reflect statistical noise due to the relatively small number of prediabetic patients and generally favourable outcome of our study cohort (i.e., a low number of adverse events). It is to be noted, that whereas prediabetic patients are at increased risk of developing cardiovascular disease, we can assume that screening and proper management might contribute to prevention of cardiovascular disease in prediabetics [4]. For example, we found a slightly higher rate of statin therapy in prediabetic than in nondiabetic patients of our study cohort. Furthermore, in our study cohort type 2 diabetes with hemodynamically significant CAD was associated with considerably worse outcome than in prediabetic patients. This makes a case for early intervention in prediabetic patients in order to prevent development of full-blown type 2 diabetes, especially in CAD patients.

Recently, the extent of coronary atherosclerosis measured as the number of segments with plaque on CTA (segment involvement score) was found to provide incremental prognostic information over stenosis severity and presence of perfusion defect in diabetic patients undergoing hybrid coronary CTA and single photon emission computerized tomography MPI [11]. Our results are generally in line with that study regarding the complementary prognostic role of anatomic and functional imaging findings in CAD; nevertheless, we did not assess the extent of coronary atherosclerosis. According to the guidelines, the selection of non-invasive tests in suspected CAD depends on, e.g., local expertise and availability, and we acknowledge that the availability of hybrid PET-CT imaging is variable [5]. The additional radiation dose from downstream PET perfusion imaging was low in our cohort, but there are also alternative methods such as CTA-based estimation of fractional flow reserve that allow non-invasive hemodynamic assessment of coronary stenosis without any additional radiation [22]. However, these were not assessed in the current study cohort, preventing direct comparison of their feasibility and prognostic value.

Notably, almost all (97%) early revascularizations in our study cohort were performed in patients with obstructive CAD associated with abnormal perfusion. Therefore, we did not include early revascularization in the multivariable models as there was a very low number of early revascularizations in other patient groups compared to those with abnormal perfusion. However, we performed sub-analyses restricted to patients not undergoing early revascularization.

Although the cohort was reasonably sized and the median follow-up time was 6.43 years, the number of adverse events remained moderately low, limiting the statistical power in subgroup analyses. Furthermore, this study was retrospective, and some information was not available. Therefore, patients with unknown diabetes status were excluded from the analysis. The registries in Finland are reliable and complete, and any detected adverse events were manually confirmed using electronic medical records [23]. All-cause mortality rather than cardiovascular mortality was used to avoid verification bias. In addition, data are missing about whether imaging triggered other secondary prevention measures than revascularizations, and regarding the control of blood glucose in patients with diabetes.

## Conclusions

Coronary CTA followed by selective downstream use of PET MPI predicts outcome in patients with suspected CAD equally in presence or absence of type 2 diabetes. In about half of the diabetic patients, obstructive CAD could be excluded by coronary CTA alone, and this was associated with favourable outcome. The prevalence of hemodynamically significant CAD was almost two-fold in type 2 diabetes compared with non-diabetic patients. The combination of hemodynamically significant CAD and type 2 diabetes was associated with the highest adverse event rate during long-term follow-up; however, there was no significant interaction between the presence of prediabetes or type 2 diabetes and the prognostic value of CTA/PET imaging findings.

## Supplementary Information

**Additional file 1**: **Table S1**. Annual event rate stratified by diabetes status and combined CTA/PET imaging findings. **Table S2**. Annual event rate stratified by diabetes status and Agatston calcium score.

**Additional file 2**: **Figure S1**. An example of a patient with multiple cardiovascular risk factors including type 2 diabetes who underwent coronary computed tomography angiography (CTA) due to exercise-related chest discomfort. There were atherosclerotic plaques with suspected obstructive stenoses (arrows) in left anterior descending (LAD; panel A) and left obtuse marginal (LOM; panel B) branches. Atherosclerotic plaques in the right coronary artery (RCA; panel C) were deemed as non-obstructive based on CTA. Due to the findings of LAD and LOM, downstream positron emission tomography (PET) myocardial perfusion imaging was performed. A polar map demonstrates moderately reduced stress myocardial blood flow in the lateral wall of the left ventricular myocardium whereas other myocardial areas are normally perfused based on PET (panel D). A fusion image of CTA and PET colocalizes the perfusion defect with LOM branch (panel E).

## Data Availability

The datasets used and/or analysed during the current study are available from the corresponding author on reasonable request.

## Abbreviations

CAD: Coronary artery disease
CTA: Computed tomography angiography
MPI: Myocardial perfusion imaging
PET: Positron emission tomography
MI: Myocardial infarction
UAP: Unstable angina pectoris
MFR: Myocardial flow reserve
ECG: Electrocardiography
ICA: Invasive coronary angiography
PCI: Percutaneous coronary intervention
CABG: Coronary artery bypass graft
ACEi: Angiotensin converting enzyme inhibitor
ARB: Angiotensin receptor blocker
