# Supplementary material for: The impact of diabetes on the relationship of coronary artery disease and outcome: a study using multimodality imaging
Source: Cardiovasc Diabetol. 2023 May 31;22:129. doi: 10.1186/s12933-023-01850-3 (PMC10230727; doi:10.1186/s12933-023-01850-3)
Supplement: Supplementary file 1 — Additional file 1: Table S1. Annual event rate stratified by diabetes status and combined CTA/PET imaging findings. Table S2. Annual event rate stratified by diabetes status and Agatston calcium score. [file 12933_2023_1850_MOESM1_ESM.docx]

**Additional file 1**

**Additional file 1: Table 1** Annual event rate (%) stratified by diabetes status and combined CTA/PET imaging findings.

| Imaging subgroup | Diabetes subgroup | Annual event rate (95% CI) | Adverse events | p-value |
| --- | --- | --- | --- | --- |
| Normal coronary CTA | No diabetes (n = 474) | 0.22 (0.10-0.46) | 7 (5 deaths, 2 MIs) | Reference |
|  | Prediabetes (n = 71) | 0.00 | 0 | N/A |
|  | Type 2 diabetes (n = 52) | 0.29 (0.04-2.07) | 1 (1 death) | 0.79 |
| Non-obstructive CAD | No diabetes (n = 388) | 1.43 (1.03-1.99) | 35 (24 deaths, 8 MIs, 3 UAPs) | Reference |
|  | Prediabetes (n = 88) | 1.48 (0.77-2.85) | 9 (8 deaths, 1 UAP) | 0.92 |
|  | Type 2 diabetes (n = 88) | 1.20 (0.57-2.51) | 7 (7 deaths) | 0.668 |
| Suspected obstructive CAD with normal Perfusion | No diabetes (n = 176) | 2.22 (1.51-3.26) | 26 (12 deaths, 12 MIs, 2 UAPs) | Reference |
|  | Prediabetes (n = 47) | 0.30 (0.04-2.09) | 1 (1 MI) | 0.048 |
|  | Type 2 diabetes (n = 58) | 2.32 (1.21-4.46) | 9 (7 deaths, 2 MIs) | 0.908 |
| Suspected obstructive CAD with abnormal perfusion | No diabetes (n = 176) | 2.57 (1.80-3.68) | 30 (17 deaths, 5 MIs, 8 UAPs) | Reference |
|  | Prediabetes (n = 53) | 2.25 (1.17-4.32) | 9 (4 deaths, 3 MIs, 2 UAPs) | 0.723 |
|  | Type 2 diabetes (n = 72) | 4.72 (3.08-7.25) | 21 (12 deaths, 8 MIs, 1 UAP) | 0.033 |

*Abbreviations as in **Table 1.**

**Additional file 1: Table S2** Annual event rate stratified by diabetes status and Agatston calcium score

| Calcium score group | Diabetes subgroup | Annual event rate (95% CI) | Adverse events | p-value |
| --- | --- | --- | --- | --- |
| Calcium score 0 | No diabetes (n = 415) | 0.25 (0.12-0.52) | 7 (6 deaths, 1 MI) | Reference |
|  | Prediabetes (n = 61) | 0.42 (0.11-1.70) | 2 (2 deaths) | 0.498 |
|  | Type 2 diabetes (n = 41) | 0.00 | 0.00 | N/A |
| Calcium score 1-99 | No diabetes (n = 283) | 1.02 (0.65-1.60) | 19 (11 deaths, 7 MIs, 1 UAP) | Reference |
|  | Prediabetes (n = 76) | 0.90 (0.38-2.17) | 5 (2 deaths, 1 MI, 2 UAPs) | 0.811 |
|  | Type 2 diabetes (n = 50) | 0.91 (0.29-2.82) | 3 (3 deaths) | 0.853 |
| Calcium score 100-399 | No diabetes (n = 194) | 1.80 (1.19-2.74) | 22 (12 deaths, 6 MIs, 4 UAPs) | Reference |
|  | Prediabetes (n = 38) | 1.05 (0.34-3.27) | 3 (3 deaths) | 0.383 |
|  | Type 2 diabetes (n = 50) | 2.45 (1.23-4.91) | 8 (7 deaths, 1 MI) | 0.455 |
| Calcium score >400 | No diabetes (n = 124) | 2.96 (1.98-4.42) | 24 (12 deaths, 6 MIs, 6 UAPs) | Reference |
|  | Prediabetes (n = 37) | 2.07 (0.86-5.00) | 5 (2 deaths, 2 MIs, 1 UAP) | 0.468 |
|  | Type 2 diabetes (n = 69) | 3.78 (2.31-6.15) | 16 (8 deaths, 7 MIs, 1 UAP) | 0.455 |

*Abbreviations as in **Table 1.**

**Figure S1.** An example of a patient with multiple cardiovascular risk factors including type 2 diabetes who underwent coronary computed tomography angiography (CTA) due to exercise-related chest discomfort. There were atherosclerotic plaques with suspected obstructive stenoses (arrows) in left anterior descending (LAD; panel A) and left obtuse marginal (LOM; panel B) branches. Atherosclerotic plaques in the right coronary artery (RCA; panel C) were deemed as non-obstructive based on CTA. Due to the findings of LAD and LOM, downstream positron emission tomography (PET) myocardial perfusion imaging was performed. A polar map demonstrates moderately reduced stress myocardial blood flow in the lateral wall of the left ventricular myocardium whereas other myocardial areas are normally perfused based on PET (panel D). A fusion image of CTA and PET colocalizes the perfusion defect with LOM branch (panel E).
