# Supplementary figures and images for: The impact of diabetes on the relationship of coronary artery disease and outcome: a study using multimodality imaging
Source: Cardiovasc Diabetol. 2023 May 31;22:129. doi: 10.1186/s12933-023-01850-3 (PMC10230727; doi:10.1186/s12933-023-01850-3)

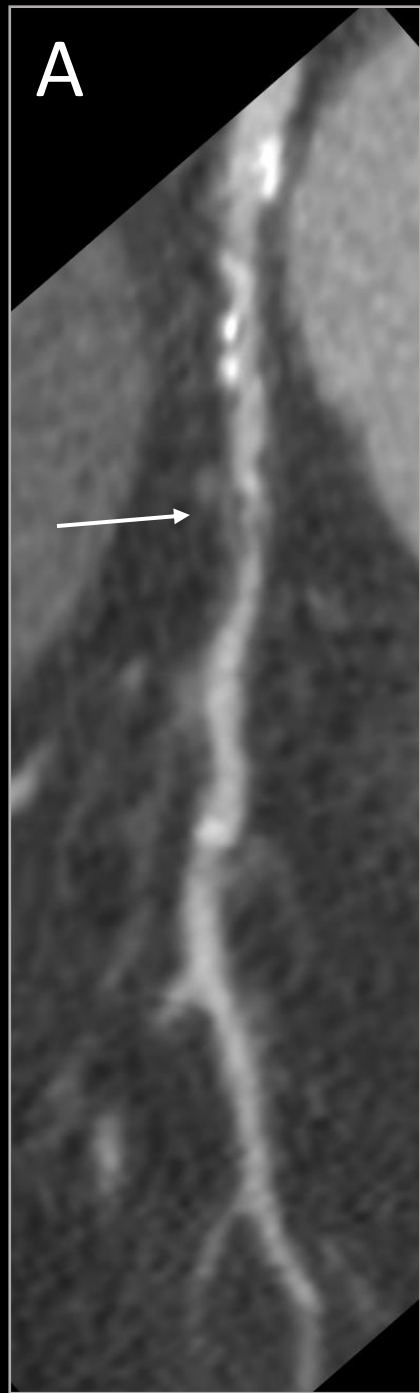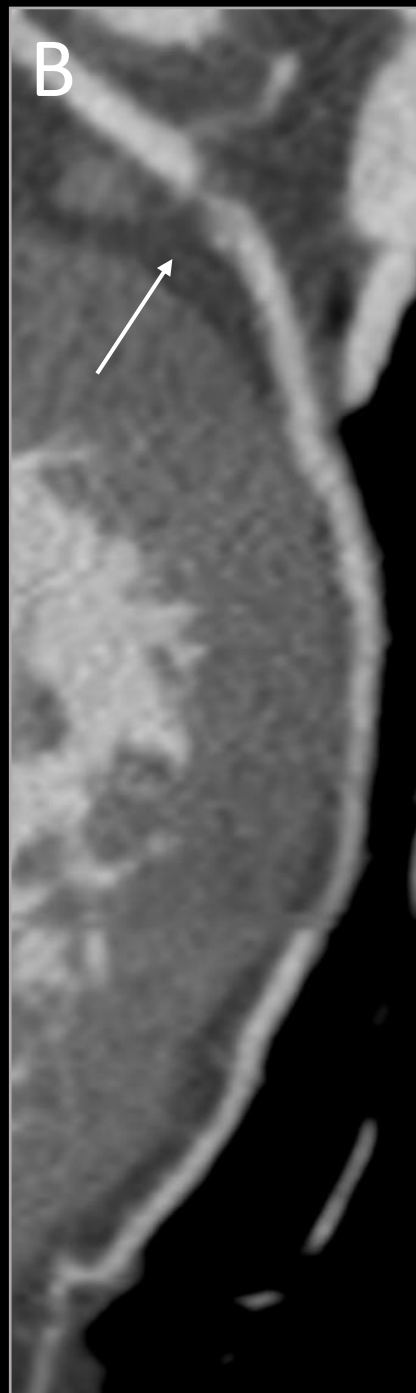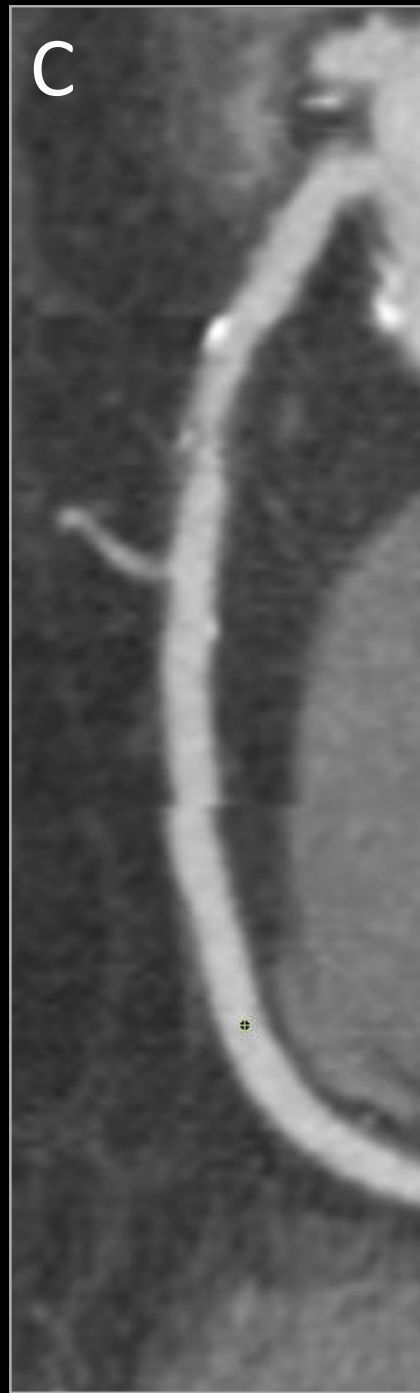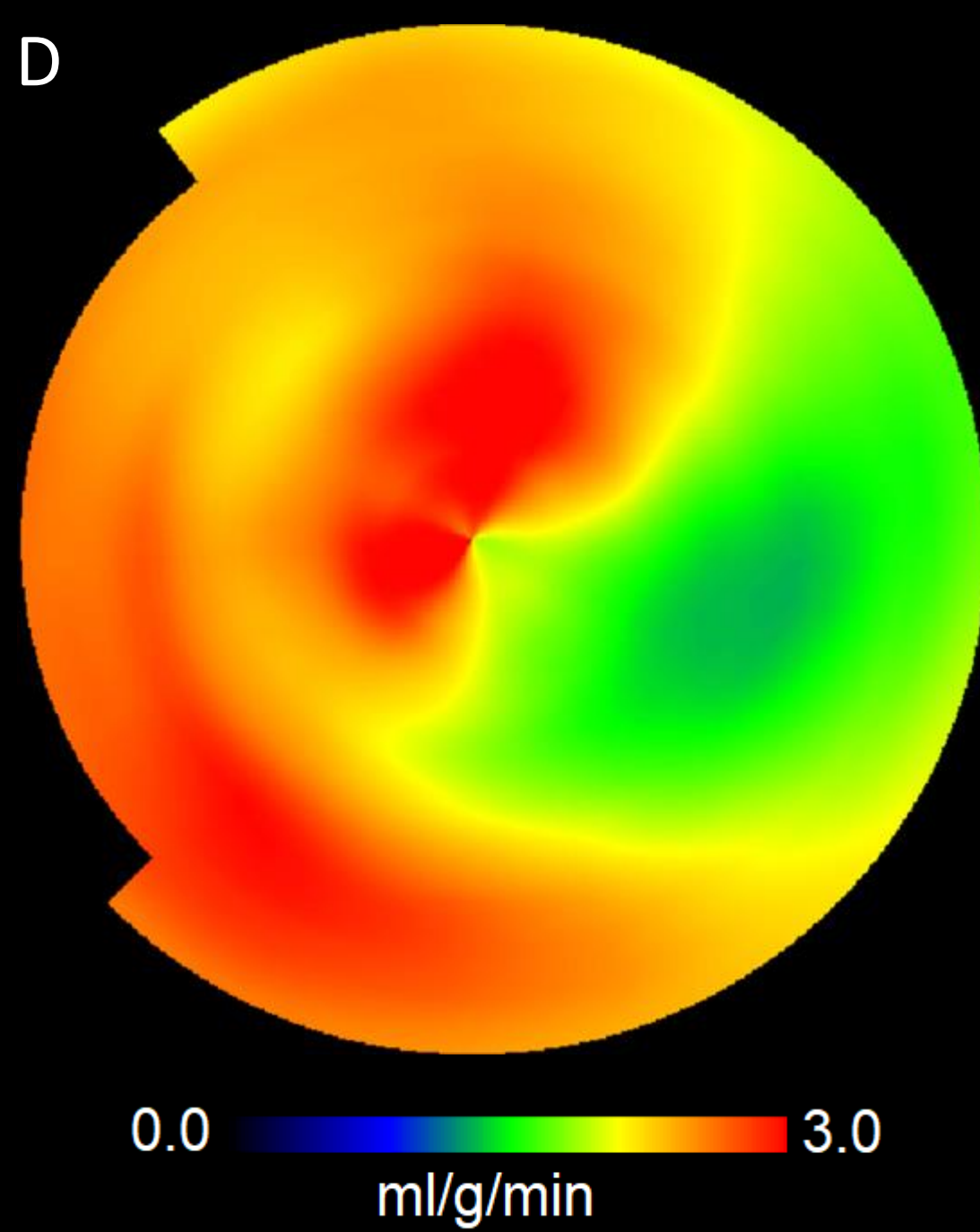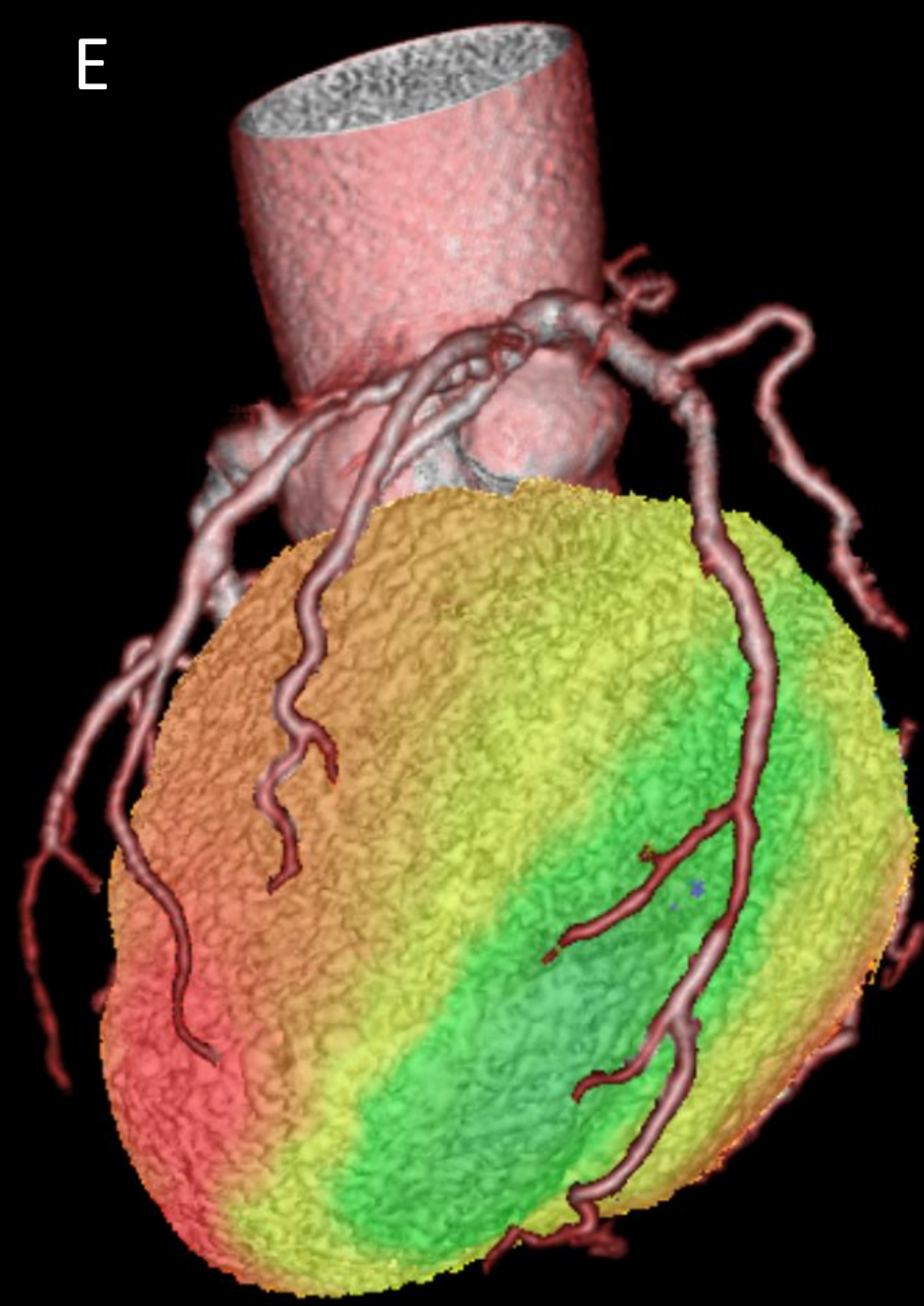

Supplement: Supplementary file 2 — Additional file 2: Figure S1. An example of a patient with multiple cardiovascular risk factors including type 2 diabetes who underwent coronary computed tomography angiography (CTA) due to exercise-related chest discomfort. There were atherosclerotic plaques with suspected obstructive stenoses (arrows) in left anterior descending (LAD; panel A) and left obtuse marginal (LOM; panel B) branches. Atherosclerotic plaques in the right coronary artery (RCA; panel C) were deemed as non-obstructive based on CTA. Due to the findings of LAD and LOM, downstream positron emission tomography (PET) myocardial perfusion imaging was performed. A polar map demonstrates moderately reduced stress myocardial blood flow in the lateral wall of the left ventricular myocardium whereas other myocardial areas are normally perfused based on PET (panel D). A fusion image of CTA and PET colocalizes the perfusion defect with LOM branch (panel E). [file 12933_2023_1850_MOESM2_ESM.pdf]
